# Predictors of Severe Course in Odontogenic Cervicofacial Infections: A Prospective Multicenter Cohort Study

**DOI:** 10.3390/jcm15176930

**Published:** 2026-09-07

**Authors:** Gian Battista Bottini, Wolfgang Hitzl, Maximilian Götzinger, Benjamin Walch, Florian Huber, Alexander Gaggl, Konstantinos Mitsimponas, Fabrizio Ferretti, Giorgia Cigna, Fabio Roccia, Christoph Steiner

**Affiliations:** 1 Department of Oral and Maxillofacial Surgery and Center for Reconstructive Surgery, Paracelsus Medical University Salzburg, Müllner Hauptstrasse 48, 5020 Salzburg, Austria; g.bottini@salk.at (G.B.B.); ma.goetzinger@salk.at (M.G.); a.gaggl@salk.at (A.G.); 2 Department of Ophthalmology and Optometry, Paracelsus Medical University Salzburg, Müllner Hauptstrasse 48, 5020 Salzburg, Austria; w.hitzl@salk.at; 3 Research Program Experimental Ophthalmology & Glaucoma Research, Paracelsus Medical University Salzburg, Müllner Hauptstrasse 48, 5020 Salzburg, Austria; 4 Department of Oral and Maxillofacial Surgery, James Cook University Hospital, Middlesbrough TS4 3BW, UK; mitsikos@yahoo.com; 5 Department of Surgical Sciences, Division of Maxillofacial Surgery, University of Turin, Città della Salute e della Scienza Hospital, c.so Dogliotti 14, 10126 Turin, Italy; fabrizio.ferretti@unito.it (F.F.); giorgia.cigna@unito.it (G.C.); fabio.roccia@unito.it (F.R.)

**Keywords:** cervicofacial infection, odontogenic infection, deep neck infection, risk factors, predictors, severe course

## Abstract

**Background:** Odontogenic cervicofacial infections are prevalent and can progress to life-threatening conditions, imposing a substantial burden on both patients and healthcare systems. The present study evaluated the association of presentation delay and comorbidities with clinical severity and healthcare resource utilization. **Methods:** This prospective multicenter cohort study enrolled patients admitted with odontogenic cervicofacial infection at two university hospitals over a one-year period. Demographic and clinical data were systematically collected. Disease severity at presentation was quantified using the Cervicofacial Infection Severity Index (CFISI), a clinically derived composite index incorporating predefined indicators of airway compromise, systemic involvement, and physiological derangement. **Results:** A total of 61 patients (median age 45 years; range 4–88) were included. The median time to admission was 4 days (IQR 2–5), and the median number of involved anatomical spaces was two (IQR 1–3). The number of involved spaces was significantly associated with a severe clinical course (OR 1.72, 95% CI 1.13–2.63, and *p* = 0.011). Secondary space involvement correlated with prolonged hospitalization, with a mean length of stay of 11.5 days (95% CI 7.1–15.9) compared to 5.1 days (95% CI 4.6–5.7) in patients without secondary space involvement (*p* < 0.00001). CFISI was significantly associated with severe clinical course in both univariable (OR 1.16, 95% CI 1.01–1.26, and *p* = 0.044) and multivariable analyses (OR 1.20, 95% CI 1.01–1.42, and *p* = 0.042). Increasing age was also independently associated with severe clinical course (OR 1.04 per year, 95% CI 1.00–1.07, and *p* = 0.028). No predictor demonstrated a significant association with high healthcare resource utilization. **Conclusions:** Within this cohort, clinical severity was associated with patient age, infection extent, and CFISI. In contrast, delay in presentation and comorbidities did not predict severe outcomes. Secondary space involvement was linked to prolonged hospitalization. No significant predictors of high healthcare resource utilization were identified.

## 1. Introduction

Odontogenic infections are the most common infections of the head and neck [1]. Pucci et al. reported 6607 visits to the emergency room (ER) of their university hospital for odontogenic abscesses over a 5-year period, accounting for approximately 10% of all ER visits [2]. A total of 151 were hospitalized, 116 underwent surgery, and 6 were in critical condition [2].

Entry points for these polymicrobial infections into the soft tissues include carious teeth, infections after dental extraction, pericoronitis in erupting teeth, and breaches in the junctional epithelium in periodontitis [3].

Once they have reached the soft tissues, they may start as cellulitis and evolve into an abscess (with pus formation) or, more rarely, into a phlegmon, mostly in immunosuppressed patients. Phlegmons can present in different forms: serous, purulent, or necrotizing; the latter is known as necrotizing fasciitis [3,4]. Cellulitis, abscess, and phlegmon all typically expand along the pathway of least resistance into potential spaces under the force of gravity [3,5]. Extension to deep cervical spaces and beyond may result in life-threatening complications (airway obstruction, mediastinitis, and sepsis) and death [3,4,6].

Flynn described the natural history of odontogenic infections as follows: edema (0–3 days), cellulitis (3–5 days), and abscess (5–7 days) [7].

History, clinical investigation, C-reactive protein (CRP) elevation, leukocytosis, and imaging confirm the diagnosis [7,8].

Limited localized infections are treated on an outpatient basis. Admission is reserved for more advanced cases. Inpatient treatment involves tooth extraction if the offending tooth is still present, incision and drainage if a pus collection has formed, and administration of intravenous high-dose broad-spectrum antibiotics. Analgesics, antipyretics, and fluids complement the surgical management. Intensive care unit (ICU) admission is required when there is an enduring threat to the airways despite incision and drainage, or there is a need for organ support [3,7].

Despite the commonality of these infections, predictors of severe courses remain incompletely defined. In the textbook *Contemporary Oral and Maxillofacial Surgery*, Flynn attributed significant weight to comorbidities, medications, and lifestyles that compromise patients’ defense against infection, such as diabetes, alcoholism, malnutrition, renal disease, chemotherapy, and immunosuppressive therapy. He advised dentists to be wary of the above risk factors in patients with dental infections [7]. Garola and associates noticed that “alcohol consumption, tobacco use, hypertension and diabetes were associated with significantly increased length of hospitalization” [9]. Färkkilä and colleagues observed that “smoking, male gender, irregular dental care and excessive alcohol consumption were significant risk factors” [10]. Tolo et al. identified age over 55 years, dyspnea, trismus, cervical involvement, and white blood cell count elevation as independent predictive factors of severe cervicofacial cellulitis [11].

Christensens et al. instead identified an increased number of involved spaces and older patient age as risk factors for reoperation in patients hospitalized for odontogenic infections [12].

Henry et al. highlighted trismus and dysphagia as risk factors for airway involvement and multispace involvement as a marker of advanced disease [1].

Es Dawoud et al. observed an association between white cell count ≥ 12 × 10^9^/L, C-reactive protein ≥ 100 mg/L, positive systemic inflammatory response syndrome criteria, lower molar pathology, and parapharyngeal space involvement with critical care admission [13].

Interestingly, Mire and colleagues found that “after adjusting for number of spaces, a primary indicator of infection severity, length of presurgical delay was not associated with either length of hospital stay (LOS) or return to the operating room”, which is a marker of a complicated course [14].

Therefore, the aim of the present study was to identify factors associated with a prolonged hospital stay (LOS) of ≥7 days, severe clinical courses, and high healthcare resource utilization.

In other words, the research question was: which factors predict a severe clinical course and demand high healthcare resource utilization in patients with odontogenic cervicofacial infections? The authors hypothesized that delayed presentation and comorbidities are key drivers of severe clinical courses, as previously suggested in the literature.

## 2. Patients and Methods

To answer the above questions, the authors conducted a prospective cohort study at their university hospitals from 1 July 2022 to 7 July 2023.

The ethics committee of the Region Salzburg granted ethical approval (number 1063/2022).

The study was conducted in accordance with the Declaration of Helsinki.

The diagnosis of odontogenic cervicofacial infection was established based on medical and dental history, clinical examination, and imaging, including panoramic radiography and, when clinically indicated, contrast-enhanced computed tomography (CT).

Patients admitted to the ward with odontogenic cervicofacial infection were enrolled after obtaining oral and written informed consent with a specific form approved by the ethics committee.

Patients with non-odontogenic infections (such as primary lymphadenopathies, skin or salivary gland infections), postoperative infections unrelated to tooth extraction, and patients who did not consent were excluded.

### 2.1. Patient-Related Variables

Demographic data (age, sex), comorbidities (diabetes, immunosuppression, and malignancy), lifestyle habits (smoking, alcohol), medications (corticosteroids, chemotherapy, immunotherapy, etc.), clinical presentation such as pain, malaise, fever, trismus, dysphagia, odynophagia, dyspnea, swelling, voice changes, signs of systemic toxicity, and the number of days between symptom onset and hospital admission were all variables in this study. A presentation was deemed delayed when 3 or more days had passed since symptom onset. “Composite immunosuppression” was defined by the presence of ≥1 of the following conditions: malignancy, steroid therapy, chemotherapy, and immunosuppressive drugs. “Comorbidity burden” was defined as the sum of the following variables: smoking, diabetes, alcoholism, composite immunosuppression, chronic cardiovascular disease, and chronic organ disease (non-cardiovascular).

### 2.2. Dental Diagnoses, Anatomic Extent, Distinction Between Primary and Secondary Spaces

Dental diagnosis, the causative tooth, and the type and number of anatomical spaces involved were recorded. The anatomical spaces were divided into primary and secondary spaces based on their proximity to the offending tooth. Primary maxillary spaces were buccal, canine, palatal, and infratemporal. Primary mandibular spaces were sublingual, buccal, and the two superficial cervical spaces: submandibular and submental. Secondary spaces were the masticatory spaces (pterygomandibular, masseteric–mandibular, deep temporal and superficial temporal), and the deep cervical spaces: parapharyngeal, carotid space, and pre-tracheal.

### 2.3. Treatment-Related Variables

Type of antibiotics, tooth extraction, incision and drainage (intraoral vs. extraoral), and need for general anesthesia are the treatment-related variables.

### 2.4. Outcomes

Length of hospital stay, admission to intensive care unit (ICU), complications, reoperations, and survival vs. death were the outcomes for this study.

### 2.5. Prolonged Length of Hospital Stay (LOS)

For the present study, a prolonged length of hospital stay (LOS) was defined as ≥7 days.

### 2.6. Severe Clinical Course

A severe clinical course was defined as the occurrence of at least one of the following events: admission to ICU, prolonged length of hospital stay (LOS)—defined as ≥7 days—occurrence of at least one acutely life-threatening complication—including Ludwig’s angina, mediastinitis, organ failure, septic shock—or other complications such as osteomyelitis or actinomycosis, failure to improve at a 48–72 h review, and in-hospital mortality.

### 2.7. Cervicofacial Infection Severity Score (CFISI)

A clinically constructed, weighted composite index, the cervicofacial infection severity index (CFISI), was developed to quantify disease severity at presentation. The CFISI is based on predefined alarm signs reflecting airway compromise, systemic involvement, and physiological derangement. Variables are weighted according to their presumed clinical severity as follows:

- Three points (major red flags): Dyspnea, impending airway compromise, impaired consciousness, neck stiffness, and tachypnea.
- Two points (moderate severity): Trismus, deviated uvula, difficulty swallowing, voice changes, appearance of illness, agitation/restlessness, severe headache, need for inpatient control of systemic disease, dehydration, tachycardia, and high fever > 38.3 °C.
- One point (supportive signs): Pallor, shivering, diaphoresis, and leukocytosis.

The CFISI score was calculated as the sum of all weighted variables present at admission, with higher scores indicating greater disease severity.

### 2.8. High Healthcare Resource Utilization

High healthcare resource utilization was defined as the occurrence of at least one of the following: Need for general anesthesia, postoperative ICU admission, or a prolonged hospital stay (≥7 days).

### 2.9. Statistical Methods

Data were checked for consistency. Fisher’s Exact test and Pearson’s Chi-Square test were used to analyze cross-tabulations. Univariable and multivariable logistic regression analyses were applied with corresponding odds ratios and 95% CIs. Relations between predictors and outcomes were illustrated using scatter and contour plots. For multivariable models, possible predictors were CFSI, composite immunosuppression, diabetes mellitus, number of anatomical spaces involved, age, sex, delay ≥ 3 days, and composite comorbidity burden. These candidate predictors were either thought to be relevant by the authors based on their clinical practice or suggested in the literature [7,11,12]. Additionally, the number of candidate variables was restricted to 8 (CFSI, composite immunosuppression, diabetes mellitus, number of anatomical spaces involved, age, sex, delay ≥ 3 days, and composite comorbidity burden) to prevent overfitting. A backward variable selection algorithm was used to further reduce the number of predictors and improve model stability. The predictors in the final model were analyzed for collinearity by computing Spearman’s correlation. The number of events per variable (EPV) was computed, and it was checked whether the standard rule (10:1) was met to prevent overfitting and increase numerical stability. All reported tests were done two-sided, and *p*-values  <  0.05 were considered statistically significant. All statistical analyses in this report were performed using STATISTICA 13 (Cloud Software Group, Inc., 2023), Tulsa, OK, USA. Data Science Workbench, version 14. Data analysis was performed as per protocol, and all statistical analyses were performed by a biostatistician (WH).

## 3. Results

### 3.1. Signs and Symptoms at Presentation

A total of 61 patients with cervicofacial infections were included in the analysis, with 34 (55.7%) males and 27 (44.3%) females. The median age was 45 years (range 4 to 88 years). A total of 49 (80.3%) patients reported painful swelling, 22 (36.1%) difficulty swallowing, 33 (54.1%) limited mouth opening, 27 (44.3%) toothache, 3 (4.9%) voice changes, and 1 (1.6%) difficulty breathing. Concerning the vital signs, 15 (24.6%) patients were tachycardic, 11 (18%) had fever, 16 (26.2%) had hypertension, and 3 (4.9%) were tachypneic. The following signs were assessed by the examining clinicians: 40 (65.6%) patients were tender to palpation, 15 (24.6%) exhibited local warmth, 17 (27.9%) erythema, 16 (26.2%) fluctuation, 14 (22,9%) enlarged cervical lymph nodes, 4 (6.5%) had a sinus tract or fistula, 53 (86.9%) a swelling, 18 (29.5%) trismus, 27 (44.3%) dental caries, 14 (22.9%) pus discharge, 3 (4.9%) deviated uvula, 10 (16.4%) a tooth tender to percussion, 31 (50.8%) dysphagia, 4 (6.5%) voice changes, 1 (1.6%) dyspnea, 5 (8.2%) impending airway compromise, 14 (22.9%) appearance of illness, 6 (9.8%) agitation and restlessness, 2 (3.3%) impaired level of consciousness, 7 (11.5%) severe headache, 3 (4.9%) neck stiffness, 3 (4.9%) pallor, 3 (4.9%) shivering, 1 (1.6%) profuse sweating, 1 (1.6%) dehydration, 5 (8.2%) decompensation of pre-existing conditions, and 44 (72.1%) had a leukocytosis.

### 3.2. Timing of Presentation vs. Severe Clinical Course and High Resource Utilization

The median time from symptom onset to hospital admission was 4 days (IQR 2–5), with a mean of 4.5 ± 4.0 days (range 1–21). A total of 68.9% of patients presented ≥3 days after symptom onset.

In univariable analysis, delay between symptom onset and hospital admission (≥3 days vs. <3 days) was not significantly associated with severe clinical course. A severe outcome was observed in 47% of patients presenting within 3 days compared to 38% of those presenting after ≥3 days (*p* = 0.57). Similarly, no significant association was found between delay and high healthcare resource utilization. High resource use occurred in 68% of early presenters and 76% of late presenters (*p* = 0.54). Overall, delay in presentation was not a significant predictor of either severe clinical course or increased healthcare resource utilization in this cohort.

### 3.3. Comorbidities vs. Severe Clinical Course and High Resource Utilization

Comorbidities were the following: malignancy in six (9.8%) patients, use of corticosteroids in three (4.9%), chemotherapy in two (3.3%), immunosuppressive drugs in one (1.6%) diabetes in seven (11.5%), hypertension in ten (16.4%), COPD in three (4.9%), smoking in twenty-two (36.1%), alcoholism in three (4.9%), schizophrenia in two (3.3%), intellectual disability in one (1.6%), renal failure, aortic aneurism and coronary disease in one (1.6%), respectively.

The authors assessed whether single comorbidities or immunosuppressive therapies were associated with a severe course or high healthcare resource utilization. However, this was not the case in the present cohort. Individual comorbidities, whether isolated or grouped, demonstrated limited, inconsistent, and often non-interpretable associations with clinical outcomes, whereas clinical severity at presentation provided a more reliable and clinically meaningful predictor. Below is a breakdown of the results.

Malignancy was associated with an increased risk of a severe clinical course (67% vs. 38%; OR 3.24, 95% CI 0.54–19.25, and *p* = 0.18), which might be significant in larger studies. Similarly, patients with malignancy more frequently required high healthcare resource utilization (83% vs. 73%; OR 1.88, 95% CI 0.20–17.39, and *p* = 0.49), but this association was not statistically significant.

Steroid therapy within the last two years was not significantly associated with severe clinical course (33% vs. 41%; OR 0.71, 95% CI 0.06–8.26, and *p* = 0.74) or high healthcare resource utilization (67% vs. 74%; OR 0.70, 95% CI 0.06–8.26, and *p* = 0.60). Interpretation is limited by the very small number of patients receiving steroid therapy.

Patients receiving cancer chemotherapeutic drugs showed a higher proportion of severe clinical course (100% vs. 39%) and high healthcare resource utilization (100% vs. 73%); however, these associations were not statistically significant (*p* = 0.16 and *p* = 0.54, respectively). Immunosuppressive drug use was observed with a higher proportion of severe clinical course (100% vs. 40%) and high healthcare resource utilization (100% vs. 73%); however, these findings are based on a single patient and were not statistically significant (*p* = 0.41 and *p* = 0.74, respectively).

Immunosuppressive comorbidities and therapies were also grouped into clinically meaningful categories to facilitate analysis and avoid over-fragmentation in multivariable models. A composite variable “immunosuppression” was defined by the presence of ≥1 of the following conditions: malignancy, steroid therapy, chemotherapy, and immunosuppressive drugs.

Composite immunosuppression was empirically associated with a higher proportion of severe clinical course (57% vs. 39%; OR 2.10, 95% CI 0.43–10.31, and *p* = 0.36), although this did not reach statistical significance. No association was observed with high healthcare resource utilization (71% vs. 74%; OR 0.88, 95% CI 0.15–5.03, and *p* = 0.88).

Diabetes mellitus was not associated with severe clinical course (43% vs. 41%; OR 1.09, 95% CI 0.22–5.36, and *p* = 0.91). A lower proportion of high healthcare resource utilization was observed among diabetic patients (43% vs. 78%; OR 0.21, 95% CI 0.04–1.09), although this did not reach statistical significance (*p* = 0.07).

Hypertension was not significantly associated with an increased risk of a severe clinical course (50% vs. 39%; OR 1.55, 95% CI 0.40–6.05, and *p* = 0.53). In contrast, hypertensive patients demonstrated a lower proportion of high healthcare resource utilization (60% vs. 76%; OR 0.46, 95% CI 0.11–1.91, and *p* = 0.28), although this finding was not statistically significant.

Coronary disease and aortic aneurysm were present in only one patient, respectively, and therefore no meaningful conclusions regarding their association with severe clinical course or high healthcare resource utilization could be drawn.

Cardiovascular conditions were also grouped into a composite “chronic cardiovascular disease”, defined by the presence of ≥1 of the following conditions: hypertension, coronary disease, and aortic aneurysm.

Composite chronic cardiovascular disease showed no significance toward an increased risk of severe clinical course (50% vs. 39%; OR 1.55, 95% CI 0.40–6.05, and *p* = 0.53). A lower proportion of high healthcare resource utilization was observed in these patients (60% vs. 76%; OR 0.46, 95% CI 0.11–1.91, and *p* = 0.28), although this was not statistically significant.

Renal failure was present in only one patient; therefore, no meaningful conclusions regarding its association with severe clinical course or high healthcare resource utilization could be drawn.

COPD/asthma was not associated with severe clinical course (33% vs. 41%; OR 0.71, 95% CI 0.06–8.26, and *p* = 0.74) or high healthcare resource utilization (67% vs. 74%; OR 0.70, 95% CI 0.06–8.26, and *p* = 0.60). Interpretation is limited by the small number of affected patients.

The composite variable “chronic organ disease” (non-cardiovascular) included the presence of ≥1 of the following comorbidities: renal failure and COPD/asthma.

Chronic organ disease was not associated with severe clinical course (33% vs. 41%; OR 0.71, 95% CI 0.06–8.26, and *p* = 0.74) or high healthcare resource utilization (67% vs. 74%; OR 0.70, 95% CI 0.06–8.26, and *p* = 0.60). Interpretation is limited by the small number of affected patients.

Smoking was not associated with severe clinical course (32% vs. 46%; OR 0.54, 95% CI 0.18–1.63, and *p* = 0.27) or high healthcare resource utilization (73% vs. 74%; OR 0.92, 95% CI 0.28–2.99, and *p* = 0.89).

Alcoholism did not show a statistically significant increased risk of severe clinical course (67% vs. 40%; OR 3.04, 95% CI 0.21–35.53, and *p* = 0.35), and no association was observed with high healthcare resource utilization (67% vs. 74%; OR 0.70, 95% CI 0.06–8.26, and *p* = 0.60). Interpretation is limited by the small number of affected patients.

Schizophrenia was present in only two patients and was not associated with a severe clinical course (50% vs. 41%; OR 1.46, 95% CI 0.09–24.47, and *p* = 0.79) or with high healthcare resource utilization (100% vs. 73%; *p* = 0.54). Interpretation is limited by the very small number of affected patients.

Intellectual disability was present in only one patient; therefore, no meaningful conclusions regarding its association with severe clinical course or high healthcare resource utilization could be drawn.

The authors defined the variable “comorbidity burden” as the total number of the following conditions per patient: smoking, diabetes mellitus, alcoholism, composite immunosuppression, chronic cardiovascular disease, and chronic organ disease (non-cardiovascular). The comorbidity burden was calculated as the sum of the above (each coded 0/1).

Comorbidity burden was not significantly associated with severe clinical course (OR 1.13, 95% CI 0.65–1.94, and *p* = 0.67) or high healthcare resource utilization (OR 0.67, 95% CI 0.37–1.21, and *p* = 0.18).

### 3.4. Dental Diagnoses

The following dental diagnoses were established: acute periapical abscess in 31 (50.8%) patients, infection after tooth extraction in 16 (26.2%) patients, acute dentoalveolar abscess in 8 (13.1%), infected remaining root fragment in 2 (3.2%), and infected odontogenic cyst and acute periodontal abscess in 1 (1.6%) patient, respectively.

The offending teeth were predominantly mandibular molars (44, 72%).

### 3.5. Treatment

A total of 44 patients (72.1%) received amoxicillin/clavulanic acid, 8 (13.1%) received clindamycin, and the remaining 9 (14.7%) received 3rd-generation cephalosporins and other antibiotics. Extraoral incision and drainage in 49 (80.3%) patients; intraoral incision and drainage in 13 (21.3%) patients; dental extraction in 39 (63.9%) patients; general anesthesia in 59 (96.7%) patients.

### 3.6. Association Between Cervicofacial Infection Severity Score (CFISI) and Risk of Severe Clinical Course

**Figure 1:**
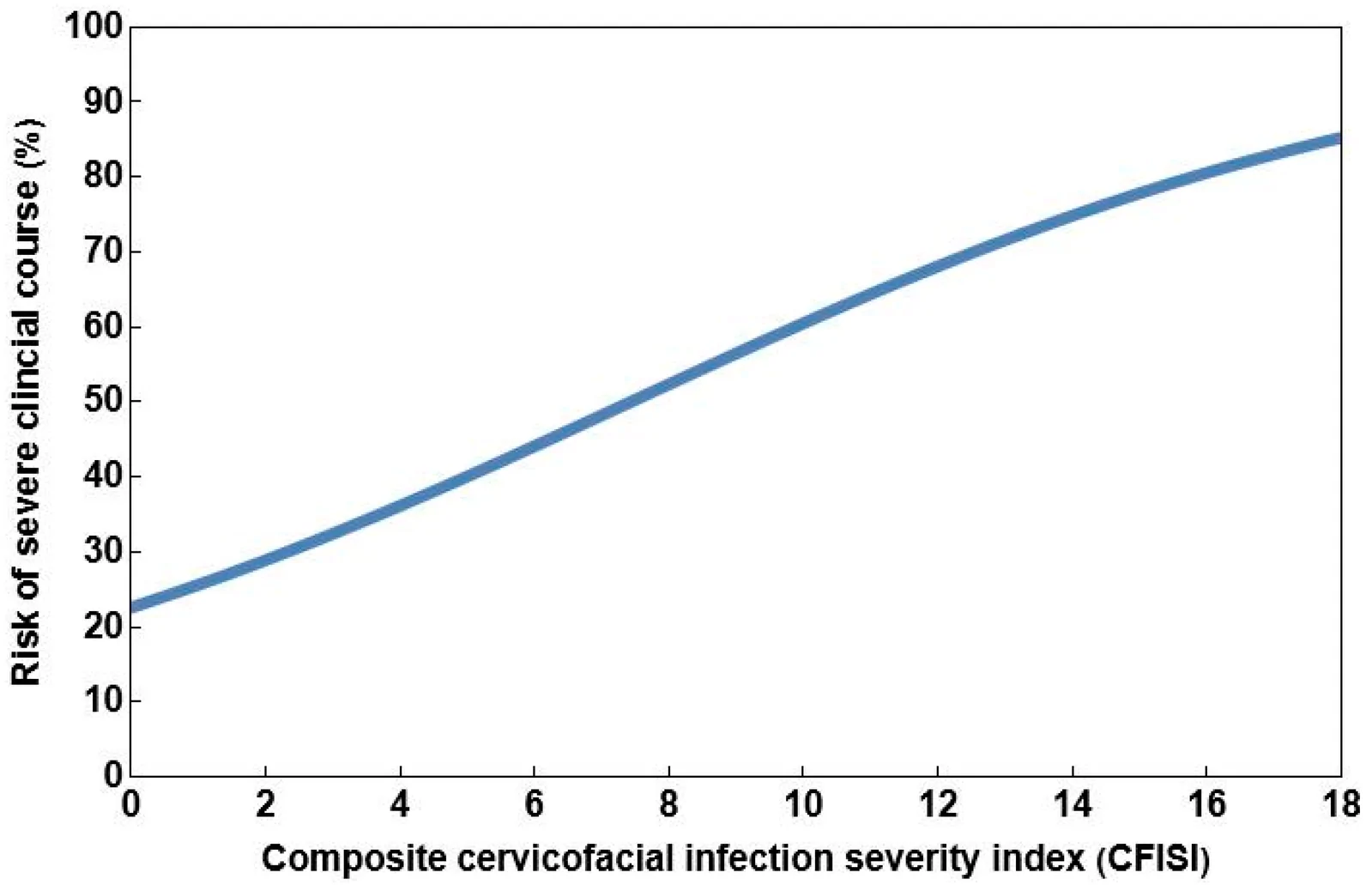
**Relation between CFISI and the risk of severe clinical course.** Line plot illustrating the predicted probability of a severe clinical course as a function of the composite cervicofacial infection severity index (CFISI) based on univariable logistic regression analysis (OR 1.16, 95% CI 1.01–1.26, and *p* = 0.044). The curve demonstrates a significant, progressive increase in risk with rising CFSI values, indicating a direct association between infection severity and adverse clinical course.

The CFISI was significantly associated with severe clinical course. In univariable logistic regression analysis, each one-point increase in CFISI was significantly associated with a 16% increase in the odds of severe outcome (OR 1.16, 95% CI 1.01–1.26, and *p* = 0.044, Figure 1). ROC analysis did not identify a clinically meaningful cutoff value, suggesting that CFISI is best interpreted as a continuous predictor of risk rather than a dichotomous variable. In contrast, no significant association was observed between CFISI and high healthcare resource utilization (*p* = 0.43).

In multivariable logistic regression analysis, including CFSI, composite immunosuppression, diabetes mellitus, number of anatomical spaces involved, age, sex, delay ≥ 3 days, and composite comorbidity burden, only age and CFSI remained significantly associated with a severe clinical course after application of a variable selection algorithm.

**Figure 2:**
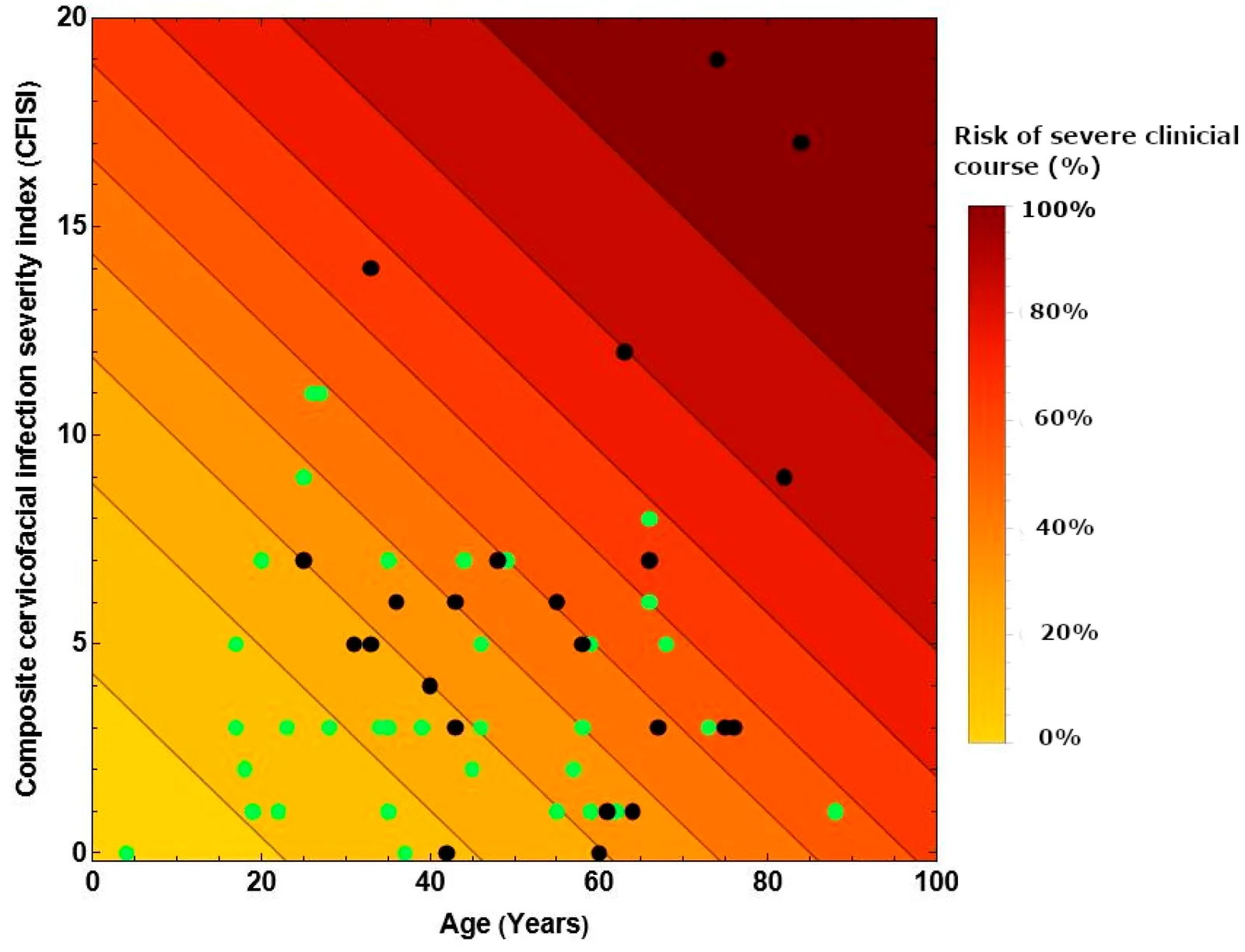
**Combined bivariable effect of age and CFISI on the risk of a severe clinical course.** CFISI was significantly associated with increased risk (OR: 1.20 per point, 95% CI 1.01–1.42, and *p* = 0.042), as was age (OR: 1.04 per year, 95% CI 1.00–1.07, and *p* = 0.028). This contour plot illustrates the predicted probability of severe clinical course based on a bivariable logistic regression model including age and CFISI. The x-axis represents age (years) and the y-axis the composite cervicofacial infection severity index (CFISI). Color gradients from yellow to red illustrate increasing risk of severe clinical course. Along diagonal contour lines with a slope of −45° representing various combinations of age and CFISI, the associated risk remains stable, demonstrating the combined effect of both variables across their respective ranges. Individual patient observations are superimposed (black dots represent patients with a severe clinical course while green dots represent patients with a standard clinical course). Age and CFSI are not correlated (r = 0.03, *p* = 0.83), suggesting no collinearity among the predictors. The events per variable (EPV) is 12, suggesting no overfitting and numerical stability of the model.

CFISI was significantly associated with increased risk (OR: 1.20 per point, 95% CI 1.01–1.42, and *p* = 0.042), as was age (OR: 1.04 per year, 95% CI 1.00–1.07, and *p* = 0.028). This relation is illustrated in Figure 2 as a contour plot. Although the odds ratio for CFISI per unit increase appeared higher than that for age, one must take the different scales of both variables into account to better interpret the effects of each predictor. The multivariable model displays contour lines with a −45° diagonal slope, indicating that age and CFISI exert a comparable influence on the risk of a severe clinical course across their respective ranges. In practical terms, younger patients with more severe infections and older patients with less severe infections may have a similar predicted risk of a severe clinical course (Figure 2). Black dots represent patients with a severe clinical course, while green dots represent patients with a standard course. The green dots are predominantly concentrated in the lower left quadrant, whereas black dots prevail in the upper-right area. Using the same predictors for high healthcare resource utilization instead of severe clinical course, no significance was found.

To illustrate its utility and applicability in clinical practice, Table 1 presents several numerical examples. For example, a 20-year-old patient with a CFISI of 10 has a 38% risk of having a severe clinical course, whereas the risk considerably increases for a 60-year-old patient with the same CFISI to 71%. An 80-year-old patient with a CFISI of 10 would have an 83% risk.

**Figure 3:**
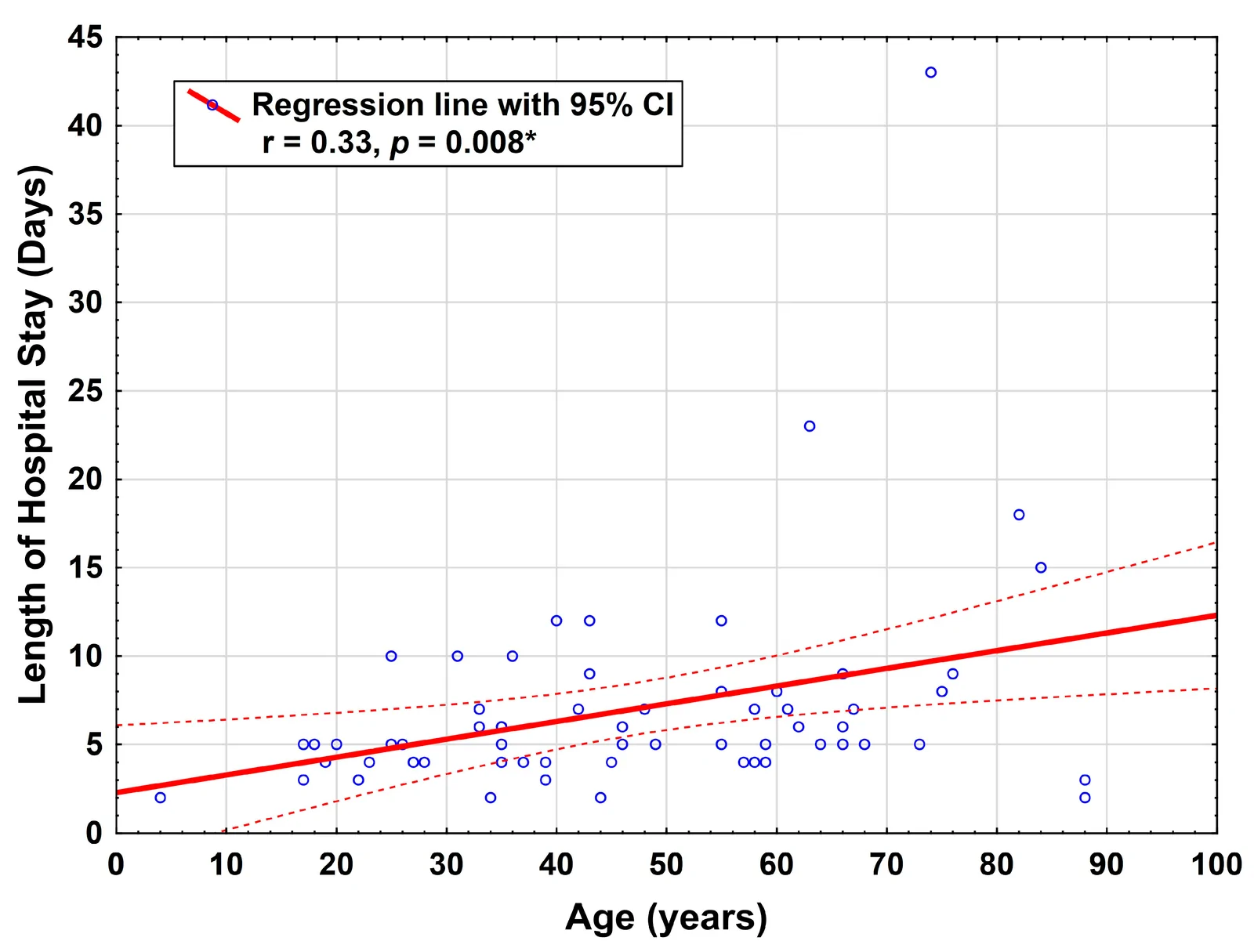
Scatter plot showing the relationship between hospital stay length (days) and patient age (years). Individual patients are represented by points, with the solid red line and dashed lines indicating the linear regression and its 95% confidence interval, respectively. * Statistically significant.

Figure 3 shows a scatter plot of hospital stay length against age, revealing a modest positive correlation with substantial variability. A slight positive association is observed (r = 0.33, *p* = 0.008), suggesting that LOS increases with advancing age, although substantial variability indicates that age alone is a limited predictor of hospital stay.

### 3.7. Fascial Spaces Involvement

The spaces involved were divided into “primary maxillary” (buccal, palatal, infratemporal, and canine), “primary mandibular” (sublingual, submandibular, and submental), and “secondary”, including the masticator spaces (pterygomandibular, masseteric–mandibular, and temporal) and the deep cervical spaces (parapharyngeal, pre-tracheal, and carotid space).

The number of involved anatomical spaces per patient was moderate, with a median of 2 (IQR: 1–3) and a mean of 2.31 ± 1.41, ranging from 0 to 6 spaces.

Most patients presented with moderate disease extent. One or two anatomical spaces were involved in 67.2% of cases, whereas 31.1% showed involvement of three or more spaces, indicating more advanced infection.

The number of involved anatomical spaces was significantly associated with severe clinical course (OR 1.72, 95% CI 1.13–2.63, and *p* = 0.011), indicating that each additional space increased the odds of severe disease.

**Figure 4:**
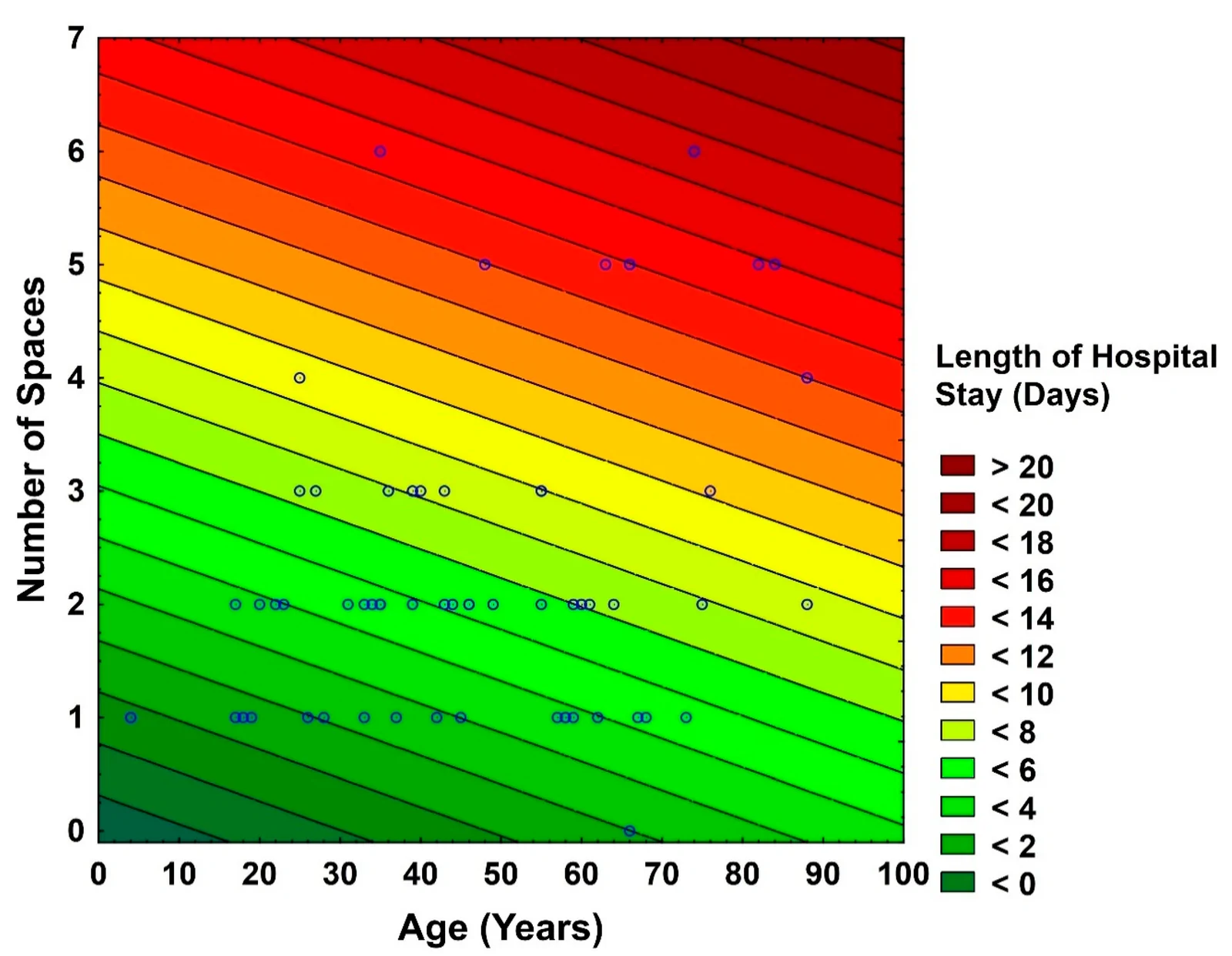
Contour plot illustrating the relation between length of hospital stay (LOS) in days, patient age, and number of involved anatomical spaces. The color gradient represents predicted LOS, ranging from shorter (green) to longer (red) hospital stays. Individual patient observations are shown as blue dots.

Figure 4 shows a contour plot of hospital stay length depending on patient age and the number of involved anatomical spaces. Hospital stay length is correlated with age (r = 0.33, *p* = 0.009) as well as the number of involved spaces (r = 0.43, *p* = 0.006). The contour lines slope more gently to the right, suggesting a more modest effect of age than of the number of spaces. In contrast, the steeper vertical gradient indicates that increasing anatomical space involvement has a substantially greater impact on prolonging hospital stay than increasing age.

**Figure 5:**
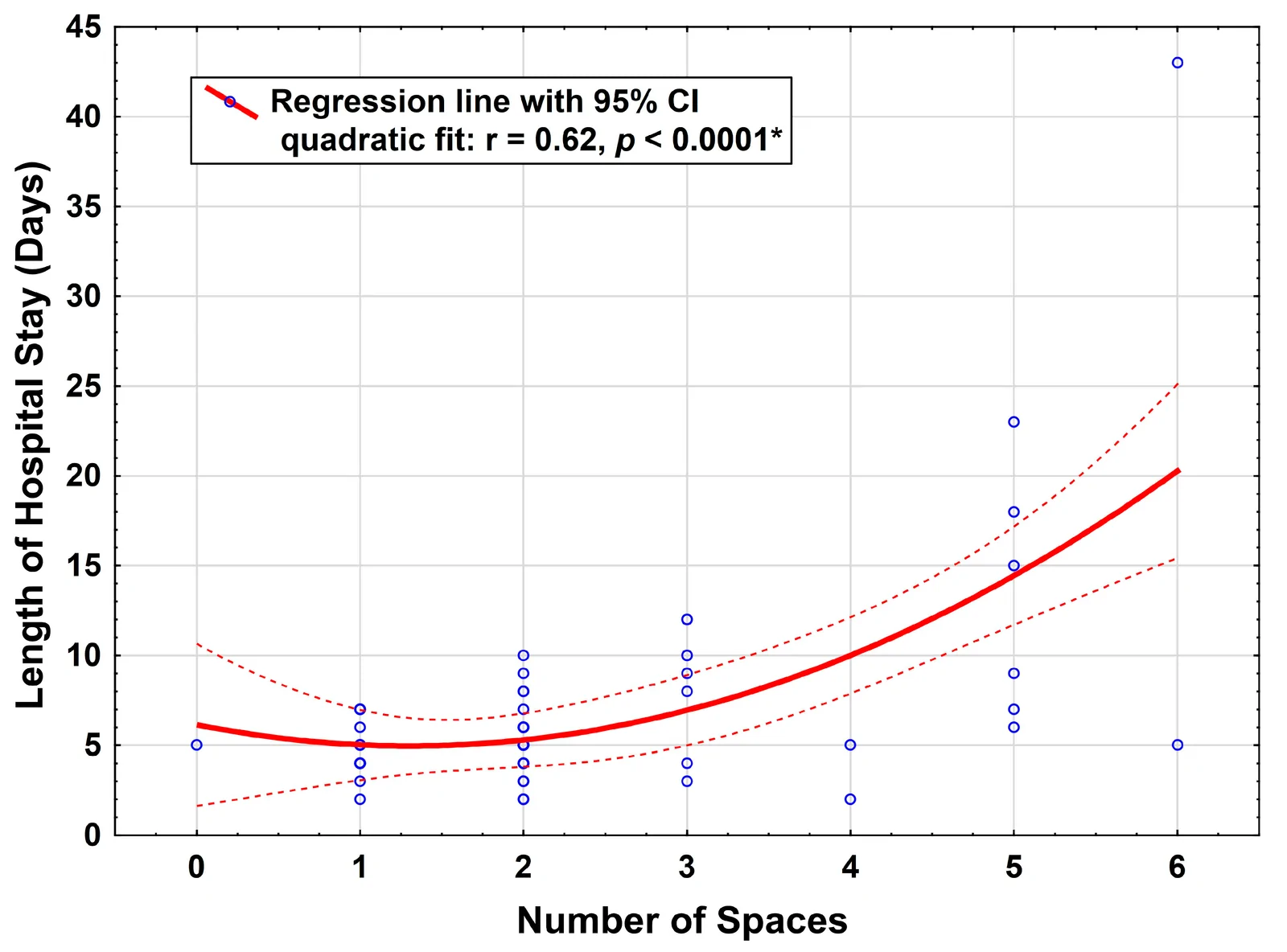
Scatter plot demonstrating a significant relation between length of hospital stay in days and number of involved anatomical spaces. Each point represents an individual patient. The solid red line indicates the fitted regression curve, with dashed lines representing confidence intervals. * Statistically significant.

Figure 5 illustrates the significant relation between hospital stay length and the number of involved anatomical spaces, demonstrating a clear increase in hospital stay with greater anatomical spread (r = 0.62, *p* < 0.0001, quadratic fit). An increasing number of involved spaces is quadratically associated with longer hospital stay and greater variability at higher levels of disease extent.

**Figure 6:**
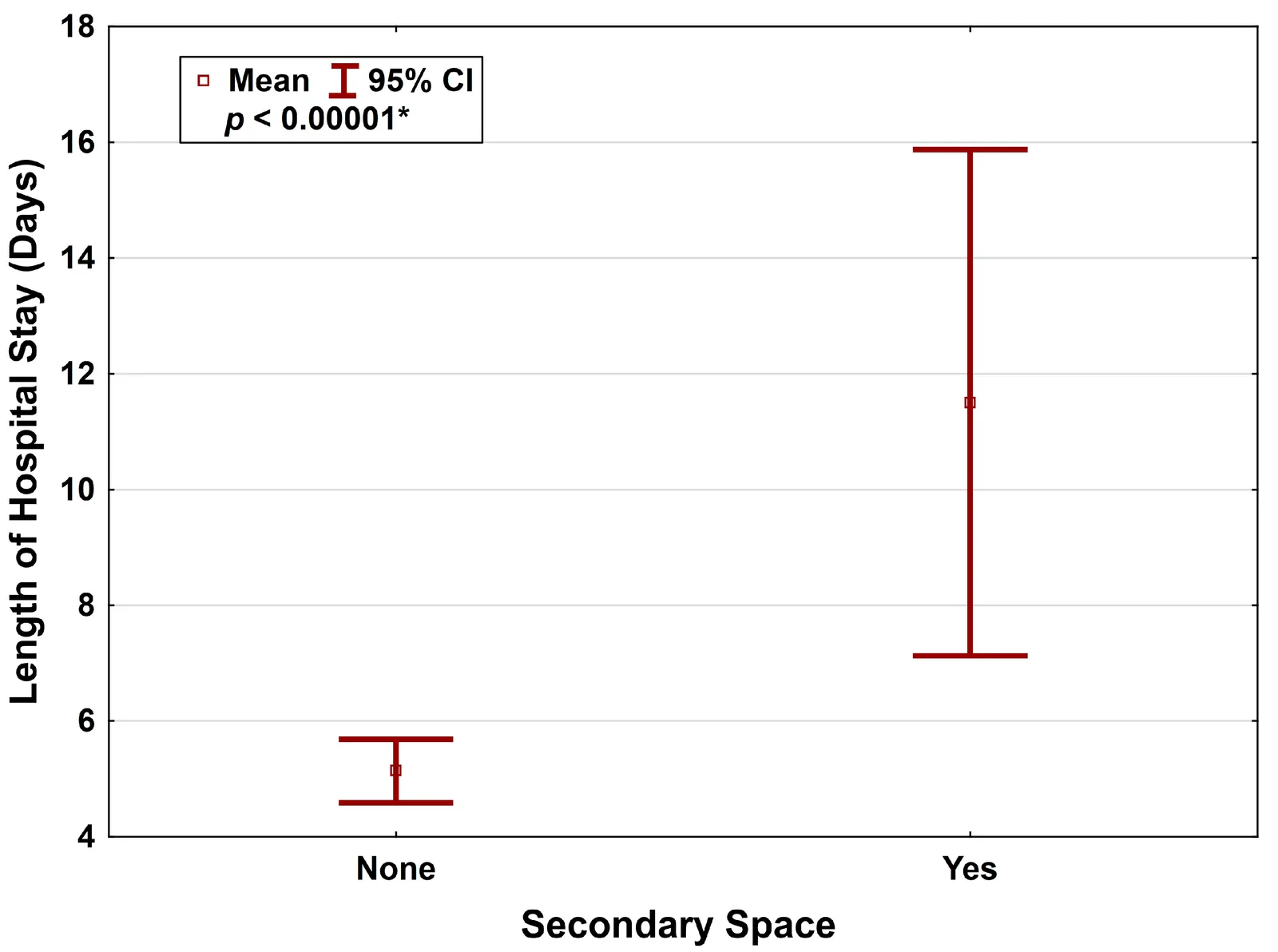
Whisker plot of the length of hospital stay according to secondary space involvement. Error bars represent 95% confidence intervals. Results are based on a generalized linear model with a log-normal distribution, *p* < 0.00001 (two-sided). * Statistically significant.

Figure 6 illustrates hospital stay length stratified by secondary space involvement, showing longer, more variable hospital stays in patients with secondary space involvement. These findings are consistent with the multivariable analysis, which identified the extent of infection (number of involved spaces) as an independent predictor of prolonged hospital stay. Patients with secondary space involvement (*n* = 18) had a significantly longer hospital stay than those without secondary space involvement (*n* = 43), with a mean length of stay of 11.5 days (95% CI 7.1–15.9) versus 5.1 days (95% CI 4.6–5.7), respectively (*p* < 0.00001).

### 3.8. Outcomes: ICU Stay, Hospital Stay, and Complications

ICU admission was required in six patients (9.8%). The median ICU stay among the six patients was 4.5 days (IQR 3–8); the mean stay was 5.2 days.

The median length of hospital stay was 5 days (IQR 4–8), with a mean of 7.0 ± 6.0 days (range 2–43), indicating a skewed distribution with a subset of patients requiring prolonged hospitalization.

**Figure 7:**
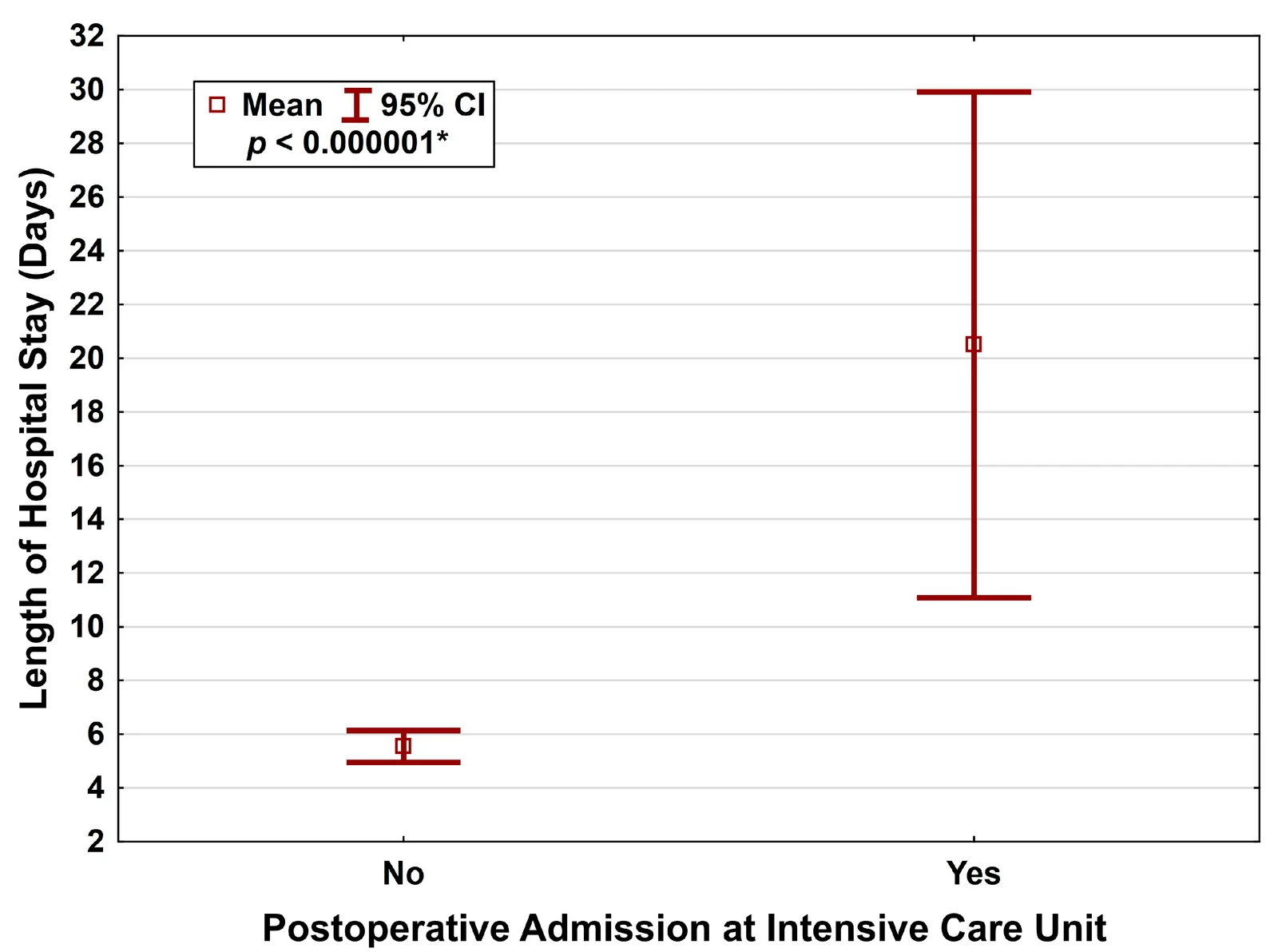
Whisker plot of length of hospital stay according to postoperative ICU admission. Error bars represent 95% confidence intervals. Generalized linear model based on a log-normal distribution, *p* < 0.000001 (two-sided). * Statistically significant.

Figure 7 shows a whisker plot of hospital stay length stratified by ICU admission, showing substantially longer, more variable hospital stays in patients requiring intensive care. Patients requiring postoperative ICU admission (*n* = 6) had a significantly longer hospital stay than those not requiring ICU admission (*n* = 55), with a mean length of stay of 20.5 days (95% CI 8.1–32.9) versus 5.5 days (95% CI 4.9–6.2), respectively (*p* < 0.000001).

Clinical improvement within 48–72 h was observed in 55 (90.2%) patients.

Sixty (98.4%) patients survived, with one in-hospital death (1.6%).

Lower jaw-related life-threatening complications included Ludwig’s angina and osteomyelitis in three patients, respectively, and descending cervical cellulitis with mediastinal involvement (one patient). No upper jaw-related life-threatening complications were recorded. Systemic complications were septic shock in three patients, delirium, kidney failure, and actinomycosis in one patient, respectively. One redo incision and drainage was necessary.

## 4. Discussion

The focus of the present study was to elucidate the relationship between delay in presentation, comorbidities, and clinical outcomes in patients with cervicofacial infections.

The findings confirm that, with adequate interventions, odontogenic cervicofacial infections generally have a favorable clinical course. The majority of patients demonstrated steady postoperative improvement and required less than six days of hospitalization. However, a clinically relevant minority (~10%) had a severe course characterized by ICU admission, delayed recovery, and prolonged hospital stay, reflecting a clear bimodal distribution of disease severity.

In accordance with the findings of Es Dawoud et al., postoperative ICU admission was strongly associated with prolonged hospitalization [13]. Patients requiring ICU care remained hospitalized approximately four times longer than those managed without ICU admission. Although ICU admission is primarily a consequence rather than a predictor of disease severity, this finding highlights the substantial clinical and organizational burden associated with severe odontogenic infections. The need for intensive care reflects the presence of airway compromise, systemic deterioration, or other serious complications requiring close monitoring and advanced supportive treatment. ICU admission not only reflects clinical severity but also serves as a surrogate marker of resource consumption, encompassing increased monitoring, staffing requirements, and prolonged inpatient care.

Among the investigated predictors, the extent of infection, expressed as the number of involved anatomical spaces, emerged as a significant determinant of severe clinical course, with each additional space markedly increasing the risk. In contrast, comorbidity burden did not show a significant association with either severity or healthcare resource utilization in this cohort, suggesting that acute disease characteristics may outweigh chronic patient-related factors in determining outcomes in this setting. Similarly, although most patients presented with a delay of ≥3 days from symptom onset, this factor alone did not appear to, per se, determine adverse outcomes, potentially reflecting the complex interplay between timing, host response, and adequate intervention. On the contrary, patients’ age correlated with prolonged LOS and with severe clinical course.

### 4.1. Delay at Presentation

Odontogenic cervicofacial infections may be conceptualized as a dynamic process that evolves over time, akin to a spreading fire. However, surprisingly, in the present analysis, delay from symptom onset to hospital admission was not independently associated with a severe clinical outcome after adjustment for confounding factors. This finding challenges the assumption that prolonged symptom duration predicts a worse clinical course and suggests that time is only one component within a complex, multifactorial process.

Extending the fire analogy, the rate of spread depends not only on time but also on the properties of the surrounding materials. Similarly, in odontogenic infections, disease progression is likely influenced by multiple interacting factors, including pathogen virulence, host immune status, and the anatomical extent and location of the infection.

Furthermore, the timing of presentation may itself be shaped by variables not captured in this study, such as symptom type, intensity, individual pain perception, and healthcare-seeking behavior, rather than reflecting the infection’s underlying severity alone. Importantly, the study did not systematically record the nature (e.g., pain versus swelling) or intensity of presenting symptoms, factors that may critically influence the decision to seek medical attention. Some patients experiencing mild or moderate symptoms may delay presentation, whereas others may seek care earlier despite a comparable extent of disease.

In this cohort, symptom duration alone appears unreliable as a surrogate for disease progression and clinical severity in odontogenic cervicofacial infections.

Our observation is corroborated by the results of Flynn et al. [15]. In a prospective case series of 37 patients, they “did not find empirical evidence to support the clinical observation that cellulitis tends to occur in the first 1 to 5 days of swelling, and that abscess formation tends to follow at 3 to 7 days”. The number of days of preoperative swelling did not significantly correlate with the presence or absence of pus found at I&D [15]. It is interesting to note that the same author described elsewhere the process of abscess development as following a predictable chronological order [7].

Also, Mire and associates did not observe a correlation between presurgical delay and LOS or reoperation [14].

### 4.2. Cervicofacial Infection Severity Index

A central finding of this study is that disease severity is the primary determinant of adverse outcome. This was consistently demonstrated across different analytical approaches. In a multivariable model including anatomical extent, the number of involved spaces emerged as the sole independent predictor of severe clinical course. Each additional space was associated with a substantial increase in risk, underscoring the importance of infection extension as a key sign of disease progression.

Further, CFISI showed a statistically significant and clinically meaningful correlation with severe clinical course, with a clear dose–response relationship. Each incremental increase in the score was associated with a higher risk of adverse outcome, supporting its validity as a measure of disease burden at presentation and as a predictor of severe clinical course.

Interestingly, no clear cutoff value was identified by ROC analysis, suggesting that risk does not abruptly increase beyond a specific threshold but rather follows a gradual continuum. This suggests that dichotomizing the CFISI may lead to the loss of clinically relevant information and that the index is better interpreted as a continuous predictor in clinical decision-making.

In a complementary model incorporating the cervicofacial infection severity index (CFISI), both CFISI and age were independently associated with severe outcome. These findings are not contradictory but rather reflect the overlapping nature of the variables. The number of involved spaces represents anatomical extent, whereas CFISI captures a broader spectrum of clinical severity, including systemic and functional parameters. Ultimately, the anatomical extent of infection and the CFSI describe the same condition—disease severity—from different perspectives.

Similarly, Es Dawoud and colleagues noticed that “combined adverse airway features (three out of four of dysphagia, trismus, stridor and voice changes) were the most accurate predictors of critical care admission” [13]. The above signs are included in the CFISI, and the results of Es Dawoud et al. further corroborate the validity of our index.

### 4.3. Anatomical Extent of Infection

In this prospective cohort, the anatomical extent of infection, quantified by the number of involved spaces, was significantly associated with prolonged hospital stay. This finding is clinically intuitive, as a wider spread of infection likely reflects a more advanced local disease process requiring more extensive treatment and monitoring. As extended hospital stay may reflect a more severe clinical course, these findings highlight both anatomical spread and age as relevant factors in the clinical trajectory of odontogenic cervicofacial infections. Both the scatterplot and contour analyses indicate an association between the number of involved anatomical spaces and the length of hospital stay, with a tendency toward longer stays with greater anatomical spread.

Secondary space involvement was associated with a significantly prolonged hospital stay. Patients with secondary space involvement had hospitalization durations more than twice as long as those without secondary space involvement, averaging 11 and 5 days, respectively. Extension of infection into secondary anatomical spaces indicates more advanced disease and requires longer healing time.

This finding is in line with the largest prospective sample published to date, which found that parapharyngeal space involvement was associated with critical care admission [13]. Consequently, secondary space involvement may serve as a practical indicator of increased healthcare resource utilization and help clinicians identify patients likely to require prolonged inpatient management.

As stated above, the number of involved anatomical spaces and the CFISI both reflect the underlying severity of infection, albeit from different perspectives. While the number of spaces represents anatomical extent, CFISI incorporates a broader range of clinical severity parameters. Accordingly, when anatomical extent was included in the model, it emerged as the dominant predictor, whereas in the model including CFISI, both CFISI and age remained independently associated with outcome. The above findings consistently indicate that, in our cohort, disease severity—whether expressed anatomically or through composite clinical indices—was the principal determinant of a severe clinical course, with age as an additional patient-related risk factor.

Likewise, Flynn et al. and Mire et al. found that multispace involvement was associated with longer LOS [14,15].

### 4.4. Comorbidities

Individual comorbidities demonstrated limited and inconsistent associations with outcome. Even clinically plausible risk factors such as diabetes mellitus, smoking, and hypertension did not show significant predictive value. While certain conditions, including malignancy and composite immunosuppression, showed trends toward an increased risk of severe clinical course, these did not reach statistical significance, likely reflecting the limited sample size. Rare comorbidities were present in too few patients to allow meaningful analysis.

Similarly, overall comorbidity burden, defined as a composite of multiple conditions, was not significantly associated with outcome in any of the models. In our sample, the immediate anatomical and clinical characteristics of the infection outweighed chronic patient-related factors in determining short-term clinical course.

Interestingly, based on our results, comorbidities did not seem to be the main drivers of complex courses. The above findings came as a surprise. Some possible explanations include: the sample size was too small to demonstrate an association; the patients with comorbidities were well-compensated and received adequate medical treatment for their chronic conditions; and the treatment for their acute dental infection was prompt and adequate.

In their series of 1002 patients, the largest prospective study published to date, Es Dawoud and colleagues did not observe an association between diabetes, age, performance status, or ASA status and ICU admission [13].

Similarly, Furuholm et al. observed that underlying disease did not predispose patients to a more severe course [16].

However, in another paper reporting on the same cohort, the same group of Es Dawoud et al. found that patients with diabetes had longer hospital stays and more returns to the operating room than non-diabetics [17].

Longer LOS in diabetic patients was also reported by Zheng et al. [18].

According to Peters 1996, comorbidities affecting the immune system were predictors of LOS [19].

Opitz et al. analyzed 14 patients with severe infections requiring ICU out of 814 hospitalized in their university hospital, observing that the 14 patients affected by severe infections “had typical predisposing factors such as diabetes mellitus, obesity, immunosuppression, and arterial hypertension. In addition, long-term alcohol and nicotine abuse and inadequate oral hygiene were noted.” [20].

Heim and colleagues, in contrast, did not observe a significant increase in LOS among diabetics in their investigation of 303 patients [21].

Søndenbroe et al. reviewed 384 hospitalized patients and noted that the proportion of patients with comorbidities and immunodeficiencies was low [22].

Boffano et al. conducted a multicenter, European, retrospective study of 6190 hospitalized patients, the largest-ever sample to assess odontogenic infections [23]. They concluded that “the presence of comorbidities was not associated with a longer hospital stay or poorer outcome.” [23].

Finally, Flynn et al. also did not find a correlation between immunocompromised patients with HIV or diabetes and LOS [15].

How do we explain the surprising and counter-intuitive finding, including the two largest multicenter series and others, that comorbidities were not significantly associated with extended LOS and complicated courses [13,15,16,23]?

We can suggest, as a hypothesis, that the medical treatment of said comorbidities has improved so much that it can effectively compensate for the significant disadvantage that immunosuppression obviously represents in fighting an infection.

### 4.5. Patient’s Age

Patient age was positively correlated with hospitalization length in the present analysis. However, age demonstrated only a modest relation with hospital stay and considerable variability, suggesting a more limited and heterogeneous effect than extent of infection. The association between increasing age and prolonged hospitalization may, at least in part, be explained by reduced physiological reserve and increased vulnerability in older patients, potentially related to frailty. However, as frailty was not directly assessed, this interpretation should be considered as a tentative explanation.

In the same line, Tolo et al. found an age above 55 to be an independent predictive factor of severe cervicofacial cellulitis [11].

Several authors observed a significantly higher LOS in older patients [14,21,23,24].

### 4.6. Resource Utilization

We could not identify predictors of high healthcare resource utilization. Ahmad et al. in 2013 attempted to put a price tag on hospitalization costs due to dental infections and reported average costs of approximately 9500 USD, ranging from approximately 1000 USD to 253,000 USD [25]. Christensen et al. in 2013 calculated an average hospital bill per patient hospitalized for odontogenic infections of approximately 17,000 USD [26]. Abramowicz and colleagues in 2013 estimated the costs of facial cellulitis of odontogenic and non-odontogenic origin to amount to “1.5 billion USD for a total of 245,000 in-hospital days for the 74,480 hospitalizations in the 2-year study period” across the USA [27]. Eisler et al. in 2012 estimated the cost of inpatient treatment for dental infections in the USA to be approximately 200 million USD per year [28].

Odontogenic cervicofacial infections could be virtually non-existent if adequate dental prevention and treatment reached the entire population. It is disheartening to observe that so many resources are deployed to treat a disease that is almost entirely preventable. Such resources in terms of personnel, operating theaters, hospital beds, and ICU beds could otherwise be allocated to other patients. Hence, it is necessary to improve oral hygiene education and preventive dental treatment across the population, especially in preschool and school-age children, to establish effective oral hygiene habits.

### 4.7. Strengths and Limitations

One strength of the present analysis is its prospective design, which enabled standardized, systematic data collection and thereby improved data quality. Second, a thorough statistical analysis was performed, enabling the identification of clinically relevant associations while accounting for potential confounding factors. Notably, the analysis also demonstrated that comorbidities were not significantly associated with severe clinical course, a finding that may appear counterintuitive but highlights the predominant role of local disease extent over systemic factors in this cohort. Finally, the identified associations are based on simple, readily assessable clinical parameters, thereby enhancing the direct applicability of the results to everyday clinical decision-making. Together, these elements strengthen the robustness, interpretability, and clinical relevance of the findings.

However, the study also has limitations that should be considered when interpreting the findings. First, the relatively small sample size may limit statistical power to detect weaker associations, particularly for secondary outcomes. In this respect, future studies with larger samples would be desirable.

Second, no significant predictors of high healthcare resource utilization were identified. This finding should be interpreted with caution, as the composite endpoint used in this study combined heterogeneous elements, including general anesthesia, ICU admission, and prolonged hospital stay, which may reflect different clinical and organizational processes. As such, the absence of association likely reflects limitations of the composite measure rather than a true lack of relationship between disease severity and resource use.

In addition, using more granular, quantitative measures of healthcare resource utilization, such as length of hospital stay and duration of ICU treatment, rather than composite binary endpoints, may enable a more precise and clinically meaningful assessment of resource consumption. Larger multicenter studies are warranted to validate these findings across different healthcare settings and patient populations.

Lastly, an additional limitation was the absence of CRP measurements in the study database. However, elevated CRP levels have already been associated with more severe courses of odontogenic infections in previous studies.

### 4.8. Clinical Implications

The findings of this study suggest that, in patients with odontogenic cervicofacial infections, precise assessment of the anatomical extent of infection should be a central component of clinical decision-making. Involvement of multiple anatomical spaces may serve as a simple, readily available marker to identify patients at increased risk of a severe clinical course who may benefit from closer monitoring, early surgical intervention, and a lower threshold for escalation of care.

In contrast, baseline comorbidity burden appears to be of limited value for short-term risk stratification in this setting.

This underscores the importance of structured clinical assessment and supports the use of composite severity indices in both clinical decision-making and research settings.

A risk assessment combining anatomical spread with clinical red flags indicating impending airway compromise, systemic involvement, and physiological derangement, together with the patient’s age, may help optimize patient outcomes while ensuring appropriate allocation of resources.

## 5. Conclusions

Based on the results of the present analysis, in odontogenic cervicofacial infections, disease severity—whether expressed as anatomical extent or composite clinical indices—is the principal determinant of adverse outcome, with patient age contributing as an additional risk factor.

## Figures and Tables

**Table 1:** Estimated risk of severe clinical course (%) using age (years) and cervicofacial infection severity index (CFISI) based on the bivariable logistic regression model in Figure 2. Individual estimated risks can be read for various combinations of age and CFSI as predictors.

Age (Years)	CFISI	Risk of Severe Clinical Course (%)
0	0	5
0	5	11
0	10	24
0	15	43
0	20	65
10	0	7
10	5	15
10	10	30
10	15	52
10	20	72
20	0	9
20	5	20
20	10	38
20	15	60
20	20	79
30	0	13
30	5	26
30	10	47
30	15	68
30	20	84
40	0	17
40	5	34
40	10	55
40	15	75
40	20	88
50	0	23
50	5	42
50	10	64
50	15	81
50	20	91
60	0	29
60	5	50
60	10	71
60	15	86
60	20	94
70	0	37
70	5	59
70	10	78
70	15	90
70	20	95
80	0	46
80	5	67
80	10	83
80	15	92
80	20	97
90	0	54
90	5	74
90	10	88
90	15	95
90	20	98

No significant predictors of high healthcare resource utilization were identified. Neither delay ≥ 3 days nor any of the assessed comorbidity or lifestyle variables showed a significant association with severe clinical course. No significant predictors of high healthcare resource utilization were identified in this model.

## Data Availability

The raw data supporting the conclusions of this article will be made available by the authors on request.
